# Incidence of and risk factors for glaucoma in lost-to-follow-up normal-tension glaucoma suspect patients

**DOI:** 10.1186/s12886-016-0245-x

**Published:** 2016-05-25

**Authors:** Jong Hoon Lim, Jun Sang Park, So Yeon Lee, Young Jae Hong

**Affiliations:** 06198 Nune Eye Hospital, #404 Seolleung-ro, Gangnam-gu, Seoul, South Korea

**Keywords:** Loss to follow-up, Normal-tension glaucoma, Glaucoma suspect, Incidence, Risk factors, Baseline IOP, Retinal nerve fiber layer

## Abstract

**Background:**

To investigate the incidence and risk factors of glaucoma in normal-tension glaucoma (NTG) suspect patients who had been lost-to-follow-up for at least 24 months.

**Methods:**

Seventy-two eyes of 72 NTG suspect patients who returned to the hospital after at least 24 months of follow-up loss were enrolled in this study between January 2009 and June 2013. The data were collected retrospectively. The incidence of glaucoma was investigated using a comprehensive glaucoma evaluation in lost-to-follow-up NTG suspect patients. The patients were classified into the glaucoma group, who developed glaucoma during the study period, and the glaucoma suspect group, who did not, to analyse the risk factors for glaucoma.

**Results:**

The number of patients who developed glaucoma was 7 (9.7 %) out of the 72 NTG suspect patients who had been mean lost-to-follow-up for 44 months. The rate of progression from suspected to glaucoma was 2.6 %/year. In the glaucoma group, the baseline intraocular pressure (IOP) was 18.43 ± 2.44 mmHg, and the average retinal nerve fiber layer (RNFL) thickness was 78.14 ± 7.60 μm; in the glaucoma suspect group, the baseline IOP was 14.95 ± 2.47 mmHg, and the average RNFL thickness was 92.55 ± 7.65 μm. The study results showed that the glaucoma group had higher baseline IOP and a thinner average RNFL (*p* = 0.003; *p* < 0.001). The results of the multivariable logistic regression analysis showed that the risk factors for glaucoma were high baseline IOP (OR = 1.63; *p* = 0.037) and a thin average RNFL (OR = 0.841; *p* = 0.004).

**Conclusions:**

The incidence of glaucoma in the lost-to-follow-up NTG suspect patients was 9.7 % for approximately 44 months, at a rate of 2.6 %/year. The risk factors for glaucoma in these patients were high baseline IOP and a thin average RNFL.

## Background

Glaucoma is a major disease that causes irrecoverable blindness worldwide [1]. Early detection and appropriate treatment are essential to preventing blindness due to glaucoma. Health check-ups and surgeries for visual acuity correction have been more common recently, and people have shown more interest in glaucoma, which has led to an increased incidence of the disease. When patients show a suspicious glaucomatous optic disc during tests for visual acuity correction surgery or during regular check-ups, they are referred to larger hospitals for more detailed tests to diagnose glaucoma. A “glaucoma suspect” is a person who has not yet developed glaucoma but has a risk of developing it in the future, characterized by consistently high IOP or abnormal optic disc, RNFL, or visual field test results that indicate suspected glaucoma [2]. Glaucoma is characterized by a morphological change in the optic disc and a subsequent functional change in visual field loss; therefore, the changes that may indicate glaucoma are followed up in glaucoma suspect patients for diagnosis confirmation. These patients must be followed up due to their risk of developing glaucoma, but they are often lost-to-follow-up because most do not manifest any symptoms or do not recognize the importance of follow-up observation and the seriousness of the disease. Most of the past large-scale epidemiological studies in populations estimated the risk of glaucoma [3–7], and some studies have investigated the incidence of glaucoma in ocular hypertension patients [8–11], but no study has been conducted to investigate the incidence of glaucoma in normal tension glaucoma (NTG) suspect patients. In this study, it was study of patients with normal tension, NTG suspect was defined as one with 21 mmHg or less IOP and with glaucomatous optic disc findings on the normal visual field test. Seventy-seven per cent of the South Korean patients with primary open-angle glaucoma (POAG) can be considered NTG [7]. As such, more studies are required on NTG suspect patients who show a high incidence of NTG in the South Korean population. No study has been conducted on the incidence and risk factors of glaucoma in lost-to-follow-up NTG suspect patients. As such, this study was conducted to analyse the incidence and risk factors of glaucoma in NTG suspect patients who had been recommended for follow-up observations without treatment but with whom contact had been lost for at least 24 months and who later returned to the hospital.

## Methods

The study protocol was reviewed and approved by the Institutional Review Board of the Nune Eye Hospital. It also strictly adhered to the principles of the Declaration of Helsinki. All subjects signed informed consent forms prior to participation.

### Study design and patients

A retrospective study was conducted using the medical records of 72 patients who visited the Nune Eye Hospital Glaucoma Centre between January 2009 and June 2013, who were diagnosed as NTG suspect patients, and who were recommended for follow-up observations without treatment, but were lost-to-follow-up for at least 24 months and later revisited the hospital. The subjects’ follow-up loss period, sex, age, glaucoma family history, and accompanying systemic disease (diabetes, hypertension) were recorded. All subjects underwent slit lamp examination for anterior segment, funduscopy, and IOP measurement using a Goldmann applanation tonometer, the manifest refraction test, central corneal thickness (CCT) measurement, optic disc stereophotography, RNFL photography, the SITA 30-2 visual field test using an automatic field analyser (Humphrey^Ⓡ^ Field Analyzer II; Carl Zeiss Meditec, Dublin, CA, USA), and average RNFL thickness measurement with optical coherence tomography (OCT; Cirrus^Ⓡ^ HD-OCT; Carl Zeiss Meditec, Dublin, CA, USA). The subjects who showed a best corrected visual acuity of 20/30 or greater, a spherical equivalent within ±6 diopter, and normal anterior segment and gonioscopy findings were enrolled in the study. Angle-closure glaucoma suspect patients are also considered glaucoma suspect patients in broad terms, but only open-angle glaucoma suspect patients with normal tension were included in this study. The patients who had ocular or neurological disorders other than glaucoma that could have affected their visual fields, who had undergone refractive surgery, and who had undergone any test for glaucoma during the lost-to-follow-up period in another hospital were excluded from this study. If both eyes were eligible for the study, one eye was randomly selected.

### Definitions

A NTG suspect was defined as one with 21 mmHg or less IOP measured two times or more using a Goldmann applanation tonometer and with glaucomatous optic disc findings on the normal visual field test. A glaucomatous optic disc is defined as one with a 0.6 or greater vertical cup/disc ratio (VCDR) or a 0.2 or greater difference in the VCDR between the eyes, minimal neural rim width <0.1 times the disc diameter or RNFL damage around the optic disc on stereophotography. Glaucoma was diagnosed if a subject showed glaucomatous visual field loss with this glaucomatous optic disc. Glaucomatous visual field loss was adjudged to exist when the threshold of three or more adjacent dots in a pattern deviation plot was 5 % or less compared with the normal, when that of one or more of the three was 1 % or less, or when that of two adjacent dots was 1 % or less on the visual field test using a Humphrey visual field analyser. In addition, findings outside of the normal limit or a 5 % or less pattern standard deviation (PSD) compared with the normal in the glaucoma hemifield test (GHT) were also equated with glaucomatous visual loss. For the visual field test, a fixation loss of 20 % or less and false negatives or positives of 15 % or less based on the reliability indices were included in the analysis. The RNFL was measured using the fast RNFL mode of OCT, and the average RNFL thickness results were used for the analysis. The OCT images with a signal strength of 6 or above with the optic disc in the centre of the scan circle were considered reliable and were included in the analysis. The average value of the three measurements of the CCT was used for the analysis. The baseline IOP was the highest IOP without using any anti-glaucoma medication at first diagnosed as NTG suspect. For the other tests index, the conventional glaucoma evaluations that were performed on the last hospital visit dates before follow-up loss were used as the baseline values. The same tests were performed upon the subjects’ hospital revisits to obtain the incidence of glaucoma. All test results were evaluated by two glaucoma specialists (J.H.L. and S.Y.L.) after analysis, and any disagreements were settled via discussion; when necessary, an additional grader (Y.J.H) was consulted.

### Statistical analysis

Statistical analysis was conducted using SPSS for Windows ver. 21.0 (SPSS Science, Chicago, IL, USA). The subjects were classified as glaucoma or glaucoma suspect depending on the development of glaucoma. The follow-up loss period, age, baseline IOP, mean deviation (MD), PSD, average RNFL thickness, VCDR, CCT, and spherical equivalent of the two groups were compared using the Mann-Whitney U-test. The chi-square test was used to compare the subjects’ sexes, glaucoma family histories, and accompanying diabetes or hypertension, optic nerve head (ONH) characteristics. Univariable logistic regression analysis was performed to analyse the risk factors for glaucoma, and the factors with *p* < 0.1 underwent multivariable logistic regression analysis. *P* < 0.05 was considered statistically significant.

## Results

There were 72 NTG suspect patients who revisited the hospital after at least 24 months of follow-up loss. Of these, 28 (38.9 %) were males and 44 (61.1 %) were females. Their mean age was 44.47 ± 12.25 years old (range: 20-73), and their mean lost-to-follow-up period was 44.76 ± 13.54 months (range: 25–84 months). Nine subjects (12.5 %) had a family history of glaucoma, and 4 (5.6 %) and 7 (9.7 %) subjects had accompanying diabetes and hypertension, respectively (Table 1). The mean baseline IOP was 15.29 ± 2.66 mmHg (range: 11–21 mmHg). The mean MD and PSD based on the visual field test were −0.88 ± 1.18 dB (range: -3.10 ~ 1.99 dB) and 1.54 ± 0.34 dB (range: 0.91–2.70 dB), respectively. The mean average RNFL thickness based on OCT was 91.15 ± 8.72 μm (range: 69–111 μm). The mean VCDR was 0.67 ± 0.06 (range: 0.50–0.90). The mean CCT and spherical equivalent were 560.32 ± 31.28 μm (range: 495–633 μm) and -1.49 ± 2.14D (range: -5.88 ~ +2.50D), respectively. We were evaluated the characteristics of ONH by stereophotography, the results are shown as a table (Table 2).

**Table 1:** Demographics of lost-to-follow-up NTG suspect patients

Parameter	Total (*n* = 72 patients)
Sex (M:F) (*n*, %)	28:44 (38.9:61.1)
Age (years)	44.47 ± 12.25
Less than 30 (*n*, %)	11 (15.3)
31–40	16 (22.2)
41–50	21 (29.2)
51–60	16 (22.2)
Over 61	8 (11.1)
Loss to follow-up period (months)	44.76 ± 13.54
Glaucoma family history (*n*, %)	9 (12.5)
Past medical history (*n*, %)	
Diabetes mellitus	4 (5.6)
Hypertension	7 (9.7)

Values are presented as mean ± SD unless otherwise indicated

**Table 2:** Clinical baseline characteristics of lost-to-follow-up NTG suspect patients

Clinical characteristics	Total (*n* = 72 eyes)
Baseline IOP (mm Hg)	15.29 ± 2.66
MD (dB)	−0.88 ± 1.18
PSD (dB)	1.54 ± 0.34
Average RNFL thickness (μm)	91.15 ± 8.72
Mean VCDR	0.67 ± 0.06
Central corneal thickness (μm)	560.32 ± 31.28
Refractive error (SE, diopter)	−1.49 ± 2.14
ONH characteristics (*n*, %)	
VCDR ≥ 0.6	48 (66.6)
VCDR asymmetry ≤ 0.2	7 (9.7)
Neural rim thinning	11 (15.3)
Focal RNFL defect	3 (4.2)
Diffuse RNFL defect	3 (4.2)

Values are presented as mean ± SD unless otherwise indicated

*IOP* intraocular pressure, *MD* mean deviation, *PSD* pattern standard deviation, *RNFL* retinal nerve fiber layer, *VCDR* vertical cup/disc ratio, *SE* spherical equivalent, *ONH* optic nerve head

The number of patients who developed glaucoma among the 72 NTG suspect patients who had been lost-to-follow-up at least 24 months in this study was 7 (9.7 %). The rate of progression from NTG suspect to glaucoma was 2.6 %/year. The mean baseline IOP in the glaucoma group was 18.43 ± 2.44 mmHg (range: 16–21 mmHg), and that of the glaucoma suspect group was 14.95 ± 2.47 mmHg (range: 11–20 mmHg), showing a significantly higher mean baseline IOP in the glaucoma group (*p* = 0.003). The average RNFL thickness was 78.14 ± 7.60 μm (range: 70–88 μm) in the glaucoma group and 92.55 ± 7.65 μm (range: 69–111 μm) in the glaucoma suspect group, showing that the glaucoma group had significantly thinner RNFLs (*p* < 0.001). The mean lost-to-follow-up period was 49.14 ± 10.90 months (range: 34–67 months) in the glaucoma group and 44.29 ± 13.79 months (range: 25–84 months) in the glaucoma suspect group, showing no statistically significant difference between the groups (*p* = 0.199). No significant differences were shown in either age, sex, glaucoma family history, accompanying diabetes or hypertension, MD, PSD, VCDR, CCT, or spherical equivalent, ONH characteristics between the groups (*p* > 0.05) (Table 3).

**Table 3:** Characteristics of lost-to-follow-up NTG suspect patients divided into two groups by developing glaucoma (glaucoma vs. glaucoma suspect)

Characteristics	Glaucoma	Suspected glaucoma	*p*-value
	(*n* = 7; 7 eyes)	(*n* = 65; 65 eyes)	
Sex (M:F) (*n*, %)	5:2 (71.4:28.6)	23:42 (35.4:64.6)	0.063^a^
Age (years)	45.00 ± 13.09	44.42 ± 12.26	0.894^b^
Loss to follow-up period (months)	49.14 ± 10.90	44.29 ± 13.79	0.199^b^
Glaucoma family history (*n*, %)	2 (28.6)	7 (10.8)	0.176^a^
Past medical history (*n*, %)			
Diabetes mellitus	1 (14.3)	3 (4.6)	0.289^a^
Hypertension	1 (14.3)	6 (9.2)	0.668^a^
Baseline IOP (mm Hg)	18.43 ± 2.44	14.95 ± 2.47	0.003^b,*^
MD (dB)	−1.01 ± 0.84	−0.87 ± 1.21	0.704^b^
PSD (dB)	1.68 ± 0.39	1.53 ± 0.34	0.296^b^
Average RNFL thickness (μm)	78.14 ± 7.60	92.55 ± 7.65	<0.001^b,*^
VCDR	0.67 ± 0.05	0.67 ± 0.07	0.774^b^
Central corneal thickness (μm)	557.00 ± 35.15	560.68 ± 31.11	0.887^b^
Refractive error (SE, diopter)	−3.06 ± 2.44	−1.32 ± 2.06	0.090^b^
ONH characteristics (*n*, %)			
VCDR ≥ 0.6	3 (42.8)	45 (69.2)	0.160^a^
VCDR asymmetry ≤ 0.2	0 (0.0)	7 (10.8)	0.361^a^
Neural rim thinning	2 (28.6)	9 (13.8)	0.304^a^
Focal RNFL defect	1 (14.3)	2 (3.1)	0.159^a^
Diffuse RNFL defect	1 (14.3)	2 (3.1)	0.159^a^

Values are presented as mean ± SD unless otherwise indicated

*IOP* intraocular pressure, *MD* mean deviation, *PSD* pattern standard deviation, *RNFL* retinal nerve fiber layer, *VCDR* vertical cup/disc ratio, *SE* spherical equivalent, *ONH* optic nerve head

^*^ *p* < 0.05 was considered significant

^a^Chi-square test; ^b^Mann-Whitney U-test

Univariable logistic regression analysis was performed to investigate the risk factors that NTG suspect patients would progress to glaucoma, and the results showed statistically significant differences between the groups in baseline IOP (*p* = 0.005) and average RNFL thickness (*p* = 0.001). In the results of the univariable logistic regression analysis, the factors with *p* < 0.1 further underwent multivariable logistic regression analysis of the relationships. Higher baseline IOP (OR = 1.63; 95 % CI: 1.03–2.57; *p* = 0.037) and thinner average RNFL (OR = 0.84; 95 % CI: 0.75–0.95; *p* = 0.004) were shown to be significant risk factors for glaucoma in NTG suspect patients (Table 4).

**Table 4:** Logistic regression analysis when the dependent variable was the presence of a glaucomatous change

Factors	Univariable analysis		Multivariable analysis^a^	
	OR (95 % CI)	*p*-value	OR (95 % CI)	*p*-value
Male gender	4.57 (0.82–25.41)	0.083		
Age (years)	1.00 (0.94–1.07)	0.904		
Loss to follow-up period (months)	1.03 (0.97–1.08)	0.371		
Glaucoma family history (*n*)	3.31 (0.54–20.41)	0.196		
Past medical history (*n*)				
Diabetes mellitus	3.44 (0.31–38.48)	0.315		
Hypertension	1.64 (0.17–15.98)	0.671		
Baseline IOP (mm Hg)	1.74 (1.19–2.56)^b^	0.005	1.63 (1.03–2.57)^b^	0.037
MD (dB)	0.90 (0.46–1.76)	0.750		
PSD (dB)	3.08 (0.40–23.53)	0.279		
Average RNFL thickness (μm)	0.83 (0.75–0.92)	0.001^b^	0.84 (0.75–0.95)^b^	0.004
VCDR	3.14 (0.00–47.13)	0.851		
Central corneal thickness (μm)	1.00 (0.97–1.02)^b^	0.766		
Refractive error (SE, diopter)	0.70 (0.49–1.00)^b^	0.053		

*OR* odds ratio, *CI* confidence interval, *IOP* intraocular pressure, *MD* mean deviation, *PSD* pattern standard deviation, *RNFL* retinal nerve fiber layer, *VCDR* vertical cup/disc ratio, *SE* spherical equivalent

^a^Backward elimination method; adjusted for all variables with *p* < 0.1 in the univariable model; ^b^OR and 95 % CI with *p* < 0.05

## Discussion

The term “glaucoma suspect” was advocated by Shaffer [12] and refers to a person with high IOP (over 21 mmHg) or an abnormal optic disc, RNFL, or visual field test results or findings [2]. A glaucoma suspect patient has not yet developed glaucoma but has the risk of developing it in the future. As such, continuous follow-up is required for the early detection of glaucoma. In an ocular hypertension treatment study (OHTS), 89 (10.9 %) of the 819 untreated ocular hypertension patients developed glaucoma at the rate of 2 %/year over 5 years [9]. Kitazawa et al. [13] conducted a study in a Japanese population and reported that 7 (9.3 %) of the 75 untreated ocular hypertension patients in their study developed glaucoma over 9 years. The incidences of progression from ocular hypertension to glaucoma varied, ranging from 0 to 35.9 %, which was considered to be related to the different subjects, observation periods, and definitions of glaucoma [8]. Kim et al. [11] reported that in their study, 24 (23.7 %) of the 101 POAG suspect patients developed glaucoma at the rate of 4.75 %/year over 5 years based on the health check-up data that they obtained. The present study was the first to have enrolled NTG suspect patients, and the study results showed that the incidence of glaucoma in the lost-to-follow-up NTG suspect patients was 7 of 72 subjects (9.7 %) over a 44-month follow-up loss period with a rate of 2.6 %/year. This rate was slightly lower than those in the previous studies that reported 3–5 %/year rates in high-risk glaucoma suspect patients [14–16]. Compared with the 0.1 %/year rate of progression to glaucoma in normal subjects based on the Melbourne Visual Impairment Project [3] and a rate of 0.55 %/year based on the Barbados Eye Study [4], the results in the present study were relatively greater.

The early detection of progression from glaucoma suspect to glaucoma patient requires regular observations. The American Academy of Ophthalmology recommends follow-up every 3–24 months depending on the patient’s risk of developing glaucoma [2]. The European Glaucoma Society also recommended follow-up every 6–12 months [17]. This study was performed in patients who had been lost-to-follow-up for at least 24 months. The mean lost-to-follow-up period was longer in the glaucoma group (49.14 months) than in the glaucoma suspect group (44.29 months), but the difference was not statistically significant. As the lost-to-follow-up period increases, patients age, and the incidence of glaucoma generally increases. As such, longer lost-to-follow-up periods will unfavourably affect the early detection of glaucoma.

Glaucoma suspect patients’ risk of developing glaucoma is known to increase with the intensity and number of risk factors [8]. The OHTS [9] and European Glaucoma Prevention Study (EGPS) [10] are typical studies on the progression from ocular hypertension to glaucoma. In both studies, high IOP, older age, thin CCT, and high PSD are considered risk factors for glaucoma. In the EGPS, a greater VCDR is considered a glaucoma risk factor. In addition, some studies reported the shape of the optic nerve, myopia, and a family history of glaucoma as risk factors for glaucoma in ocular hypertension [18–20]. Kim et al. [11] suggested that old age, high baseline IOP, high BMI, high level of education, and high hematocrit level were risk factors for glaucoma for POAG suspect patients.

The results of the univariable logistic regression analysis that was performed in this study to identify the risk factors for glaucoma in NTG suspect patients showed that there were statistically significant differences in baseline IOP (OR = 1.74; *p* = 0.005) and in average RNFL thickness (OR = 0.83; *p* = 0.005) between the glaucoma and glaucoma suspect groups. Multivariable logistic regression analysis was performed using the factors with *p* < 0.1 in the univariable logistic regression analysis. The results also showed that high baseline IOP (OR = 1.63; *p* = 0.037) and a thin RNFL (OR = 0.84; *p* = 0.004) were significant risk factors for glaucoma in NTG suspect patients.

The differences between the glaucoma risk factors identified in this study and those identified in the previous studies were considered to be due to the different subject groups, including different ethnicities; other differences were attributed to selection bias, different diagnosis methods, different definitions of glaucoma, different statistical analysis methods, and differences in numbers of subjects. Both the OHTS and the EGPS were conducted in ocular hypertension patients, whereas this study was conducted in NTG suspect patients. Old age was not considered a glaucoma risk factor in this study, although it was considered to be so in many previous studies. This finding was attributed to the relative youth (mean age: 44.47 years) of the subjects in this study and to the fact that only 8 subjects (11.1 %) were 61 years old or older. More systematic studies with a greater number of subjects are required in the future.

High IOP was considered the most important risk factor for glaucoma. Many previous studies on the incidence of glaucoma indicated that patients with higher IOP showed higher incidences of glaucoma [3–7]. In this study, the baseline IOP was significantly higher in the glaucoma group (18.43 mmHg) than in the glaucoma suspect group (14.95 mmHg), and the results of the multivariable logistic regression analysis showed that high baseline IOP was a glaucoma risk factor in the NTG suspect group (OR = 1.63; *p* = 0.037), which was similar to the results of the previous studies [9–11]. The role of IOP as part of the NTG aetiology has been controversial, but the results of this study support the important role of high IOP in developing glaucoma.

In glaucoma, the RNFL of the optic nerve is known to become progressively thinner with the loss of retinal ganglion cells. Therefore, RNFL thickness is considered important in the diagnosis and follow-up of the progression to glaucoma [21, 22]. Glaucoma shows a change in the optic disc and RNFL prior to any functional damage, such as changes in the visual field. It is known that decreased RNFL thickness comes before a change in the optic disc [22–25]. One study reported that the mean RNFL thickness among Koreans was 100.84 μm [26]. Hirasawa et al. [27] reported a mean RNFL thickness of 102 μm in 251 normal eyes of Japanese participants measured using SD-OCT. The average RNFL thickness in Asian populations has been reported to be 100 μm or more [28]. This study showed an average RNFL thickness of 91.15 μm in NTG suspect patients and showed that the average RNFL thickness in the glaucoma group (78.14 μm) was significantly thinner than that in the glaucoma suspect group (92.55 μm). Furthermore, the multivariable logistic regression analysis showed that a thin average RNFL could be a risk factor for glaucoma in NTG suspect patients (OR = 0.84; *p* = 0.004).

This study had some advantages compared with the previous population-based studies: (1) it was the first study that was conducted in lost-to-follow-up NTG patients; (2) it minimized investigator bias because the subjects were lost-to-follow-up on their own volition; (3) various ocular tests could be conducted because the study was hospital- rather than population-based and, accordingly, the ophthalmological risk factors could be analysed.

This study, however, also had a number of limitations. First, the data were analysed retrospectively using the subjects’ medical records, and many of the data on loss to follow-up were dependent on the subjects’ descriptions, which made the data less objective. In addition, the incidence of glaucoma requires the long-term investigation of an extensive population, but this study had a limited number of subjects, and the lost-to-follow-up period was too short for identifying glaucoma risk factors. Considering the conditions of subject enrolment, however, only the patients who were diagnosed as NTG suspects, who had been lost-to-follow-up for some time, and who had revisited the hospital were enrolled in the study, making it difficult to recruit subjects.

## Conclusions

This study was conducted to identify the incidence and risk factors of glaucoma in NTG suspect patients according to the lost-to-follow-up period and to draw attention to the implications of being lost-to-follow-up. In conclusion, approximately 9.7 % of the lost-to-follow-up NTG suspect cases in this study developed glaucoma over 44 months, and the progression rate was 2.6 %/year. This result emphasizes the importance of regular follow-up observation through thorough patient training given that the NTG suspect patients in this study showed a higher incidence of glaucoma compared with the healthy participants. In addition, NTG suspect patients with confirmed risk factors such as high baseline IOP and a thin average RNFL should be more carefully observed.

## Abbreviations

CCT, central corneal thickness; EGPS, European glaucoma prevention study; IOP, intraocular pressure; MD, mean deviation; NTG, normal-tension glaucoma; OCT, optical coherence tomography; OHTS, ocular hypertension study; ONH, optic nerve head; OR, odds ratio; POAG, primary open-angle glaucoma; PSD, pattern standard deviation; RNFL, retinal nerve fiber layer; SD, standard deviation; SE, spherical equivalent; VCDR, vertical cup/disc ratio.
